# Shining a Special Light on an Unusual Case of Thoracic Lymphadenopathy

**DOI:** 10.7759/cureus.89276

**Published:** 2025-08-03

**Authors:** Abououf Marwan, Sarah Nicholson, Omar Elboraey, Maged Hassan

**Affiliations:** 1 Pulmonology, Royal Preston Hospital, Preston, GBR; 2 Pathology, Lancashire Teaching Hospitals NHS Foundation Trust, Preston, GBR; 3 Respiratory Medicine, Mersey and West Lancashire Teaching Hospitals NHS Trust, Preston, GBR; 4 Chest Diseases, Alexandria University Faculty of Medicine, Alexandria, EGY

**Keywords:** amyloidosis, amyloid plaques, congo red, lymphadenopathy, mediastinal

## Abstract

Amyloidosis is an uncommon disease characterized by the buildup of misfolded protein fibrils outside cells in various tissues. Its diagnosis relies on tissue biopsy confirmation. Due to its diverse clinical presentations, diagnosis is often delayed. The symptoms depend on the organs affected, with the kidney and heart being most frequently involved. Although pulmonary involvement occurs relatively often, it seldom causes symptoms. Pulmonary amyloidosis can manifest as nodular pulmonary amyloidosis, diffuse alveolar-septal amyloidosis, or tracheobronchial amyloidosis. This case highlights a rare incidental finding of isolated amyloid lymphadenopathy without additional pleuropulmonary disease.

## Introduction

Systemic amyloidosis is an infrequent cause of mediastinal lymphadenopathy [1]. Its classification depends on the type of protein deposited, disease origin (primary, secondary, or hereditary), and whether the condition is systemic or localized. Patients with localized amyloidosis in the chest typically present with pulmonary nodules or laryngotracheobronchial lesions, whereas lymphadenopathy is rarely observed in localized cases [1]. This case highlights a rare presentation of systemic amyloidosis, diagnosed incidentally after the detection of isolated mediastinal lymphadenopathy, without other pulmonary manifestations.

## Case presentation

A 75-year-old male presented to the emergency department with palpitations, chest pain, and dizziness. He had a history of a previous similar episode three days earlier with loss of consciousness and a fall, which left him with multiple left-sided bruises and shoulder pain. He denied shortness of breath, fever, or night sweats. However, he reported a marked decline in his exercise tolerance, from one mile to 50 yards, over a few months preceding admission. His medical history included atrial fibrillation, chronic obstructive pulmonary disease, and monoclonal gammopathy of undetermined significance (MGUS) for which he was not under regular follow-up.

Physical examination revealed bruising to the lateral aspect of the 6th to 10th ribs on the left side with tachypnoea of 28 breaths per minute. He also had bruises on the right hip with a limited range of motion to only 20 degrees on the straight leg test due to pain. Additionally, bilateral pedal edema was noted up to the mid-calf, which the patient reported to be of recent onset, having only started a month earlier. The rest of the examination was unremarkable, and vital signs were within normal range.

After taking a detailed history and arranging relevant investigations, the multiple falls were linked to orthostatic hypotension.

Routine blood investigations were unremarkable. The white cell count (WCC) was 9.52 × 10⁹/L, hemoglobin (Hb) was 159 g/L, and platelet count was 280 × 10⁹/L. Renal function tests revealed a urea level of 5.5 mmol/L and a serum creatinine of 75 µmol/L. Liver function tests were within normal limits. An electrocardiogram (ECG) demonstrated a normal sinus rhythm.

**Figure 1:**
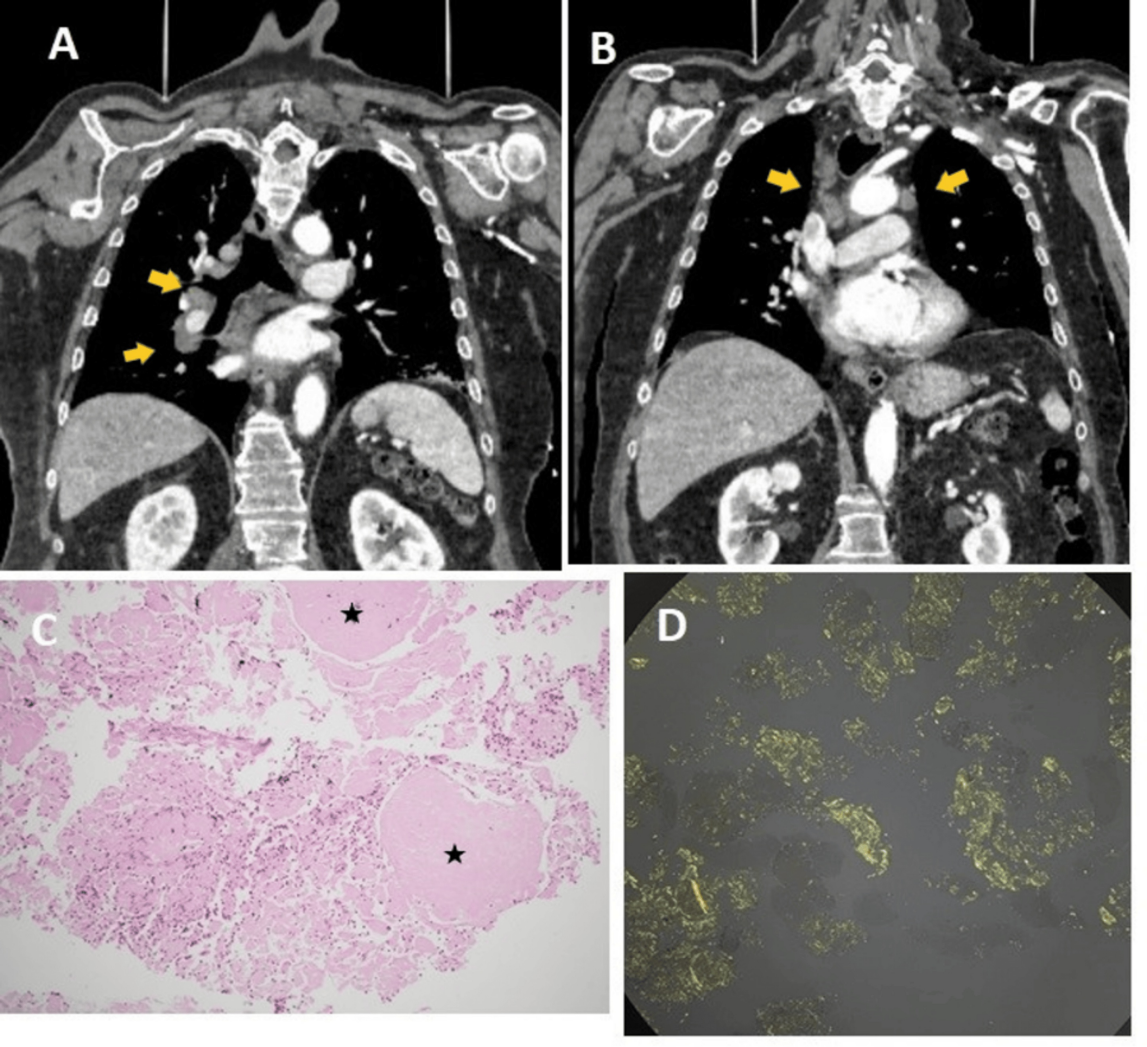
Panels A and B: Coronal section of the CT scan of the thorax showing enlarged hilar and mediastinal lymph nodes (yellow arrows). Panel C: H&E staining showing inflammatory cells surrounding areas of homogeneous acellular eosinophilic material (asterisks). Panel D: Congo red-stained slide examined under polarized light showing the characteristic apple-green birefringence.

To exclude bone fractures, a trauma scan was arranged, which revealed no internal injury but identified enlarged mediastinal lymph nodes (arrows in Figures 1A, 1B): an enlarged right hilar lymph node measuring approximately 25 mm and a right paratracheal lymph node measuring approximately 20 mm.

Bronchoscopic endobronchial ultrasound (EBUS)-guided biopsy of the subcarinal and right paratracheal lymph nodes was performed. Hematoxylin and eosin staining revealed amorphous eosinophilic material with mixed inflammatory cells (asterisks in Figure 1C).

Congo red staining observed under polarized light exhibited the characteristic apple-green birefringence indicative of amyloid deposits (Figure 1D).

Additional workup demonstrated IgM lambda paraproteinemia and nephrotic-range proteinuria (urine polymerase chain reaction: 430 mg/mmol). Kidney biopsy confirmed amyloid deposits, and immunohistochemistry revealed IgM and lambda light chains. A diagnosis of systemic light chain (AL) amyloidosis was established. The patient was started on treatment with rituximab and bendamustine, but it was discontinued after three cycles due to adverse effects, as the patient had multiple hospitalizations with postural hypotension and acute kidney injury (AKI).

At 12-month follow-up, he remained under hematology care and commenced regular hemodialysis for progressive renal failure.

## Discussion

Systemic AL amyloidosis results from abnormal protein folding, causing widespread organ damage [2]. Monoclonal gammopathies and myeloma are common predisposing conditions. The disease most often affects the kidneys, heart, and autonomic nervous system.

Respiratory manifestations typically include pleural effusions [2], with other features such as pulmonary nodules, diffuse alveolar infiltrates, tracheobronchial nodules, and intrathoracic lymphadenopathy [3]. However, isolated lymphadenopathy without other pulmonary involvement, as in this case, is rare [4]. Stem cell transplantation (SCT) offers one of the most effective treatments, with average remission exceeding 10 years, though less than 20% of newly diagnosed patients qualify due to extensive organ involvement and poor health status [2].

In the workup of patients with isolated mediastinal lymphadenopathy, no features on computed tomography or EBUS examination are suggestive of amyloid deposition. Transbronchial fine needle aspiration is a useful and safe procedure for sampling mediastinal lymph nodes [5]. The presence of homogeneous eosinophilic material on routine histology can raise suspicion, but definitive diagnosis requires Congo red staining examined under polarized light to demonstrate the pathognomonic apple-green birefringence.

## Conclusions

This case illustrates a rare presentation of AL amyloidosis manifesting as isolated mediastinal lymphadenopathy, later complicated by renal involvement. It emphasizes the importance of considering amyloidosis in the differential diagnosis of mediastinal lymphadenopathy, particularly in patients with underlying MGUS. Although the disease progressed to end-stage renal disease necessitating renal replacement therapy in this patient, early diagnosis and multidisciplinary management remain critical to improving outcomes in amyloidosis.
